# AS1041, a Novel Synthesized Derivative of Marine Natural Compound Aspergiolide A, Arrests Cell Cycle, Induces Apoptosis, and Inhibits ERK Activation in K562 Cells

**DOI:** 10.3390/md15110346

**Published:** 2017-11-04

**Authors:** Fengli Yuan, Liang Qiao, Yinghan Chen, Xin Qi, Yankai Liu, Dehai Li, Qianqun Gu, Jing Li, Ming Liu

**Affiliations:** 1 Key Laboratory of Marine Drugs, Ministry of Education, School of Medicine and Pharmacy, Ocean University of China, Qingdao 266003, China; ygg20113379@163.com (F.Y.); liang.q@yahoo.com (L.Q.); qd_yinghan@163.com (Y.C.); qxhin@163.com (X.Q.); liuyankai@ouc.edu.cn (Y.L.); dehaili@ouc.edu.cn (D.L.); guqianq@ouc.edu.cn (Q.G.); 2 Laboratory for Marine Drugs and Bioproducts of Qingdao National Laboratory for Marine Science and Technology, Qingdao 266237, China

**Keywords:** aspergiolide A derivatives, apoptosis, cytotoxicity, ERK, topoisomerases

## Abstract

AS1041 is a novel synthesized anthraquinone lactone derivative of marine natural compound aspergiolide A (ASP-A) with new structure skeleton and marked cytotoxicity in cancer cells. To study its cytotoxicity in detail, we evaluated its activity on human K562 chronic myelogenous leukemia cells and investigated the related molecule mechanisms. AS1041 significantly inhibited the proliferation and colony formation of K562 cells. Moreover, AS1041 arrested cell cycle progression at G2/M phase in a concentration-dependent manner, and also caused concentration- and time-dependent induction of apoptosis. In addition, the molecular mechanisms investigation showed that AS1041 did not localize in the cellular nucleus and did not affect topoisomerases I or II. However, AS1041 could inactivate extracellular signal-regulated kinase (ERK) and contribute to AS1041-induced apoptosis. We concluded that AS1041 was cytotoxic to K562 leukemia cells and the cytotoxicity related to the cell cycle arrest, apoptosis induction, and ERK inhibition. These results implied that AS1041 was a novel derivative of ASP-A with significant cytotoxicity to chronic myelogenous leukemia cells and may have therapeutic potential for the treatment of cancer and leukemia.

## 1. Introduction

Cancer remains a vast disease worldwide. Natural products, or their derivatives, represent more than half of the clinical cancer chemotherapeutic agents, such as etoposide, taxanes, and camptothecines [1,2,3]. Due to the special chemical and physical conditions of marine ecology, marine organisms biosynthesize a variety of molecules with unique chemical structures. These compounds provide multiple and potent pharmacological activities [4]. Most notably, these marine organism-originated compounds, and their derivatives, are an important source for the discovery of anticancer agents [5], and an increasing number of marine natural compounds or their synthesized derivatives have been proved to possess potent anticancer activities, with the most potent class of compounds have entered the clinical trials, or even clinical use [6]. The great progress in the anticancer area using marine drugs make us believe that it is a valid method to search anticancer drugs from marine natural compounds or their synthesized derivatives.

**Figure 1:**
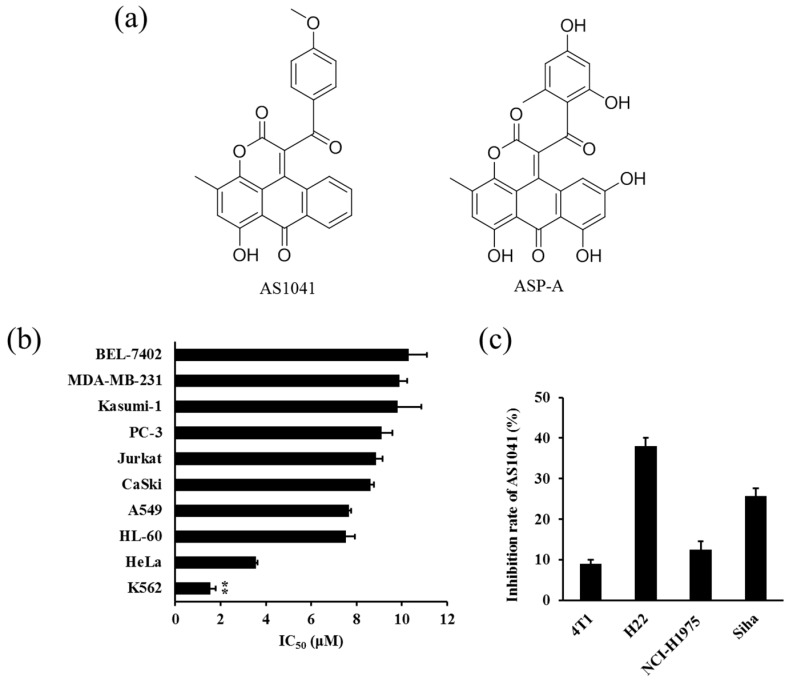
Cytotoxic effect of AS1041. (**a**) Chemical structure of AS1041 and aspergiolide A (ASP-A). (**b**) IC_50_ values of AS1041 on selected human cancer cells (K562, HeLa, HL-60, A549, CaSki, Jurkat, PC-3, Kasumi-1, MDA-MB-231, and BEL-7402). Cells were treated with AS1041 for 72 h. Cell viabilities were examined by the 3-(4,5-dimethylthiazol-2-yl)-2,5-diphenyltetrazolium bromide (MTT) or sulforhodamine B (SRB) assay. ** *p* < 0.01 vs. other cell lines. (**c**) Inhibition of AS1041 on 4T1, H22, NCI-H1975, and Siha cells. Cells were treated with AS1041 (10 μM) for 72 h. Cell viabilities were examined by MTT or SRB assay. Data are presented as mean ± SD for three independent experiments.

Novel anthraquinone lactone compound AS1041 (Figure 1a left) is one of the synthesized derivatives of aspergiolide A (ASP-A, Figure 1a right) which possesses a novel structure skeleton [7]. Our preliminary work displayed that ASP-A, isolated from marine fungus *Aspergillus glaucus,* had anticancer effect via inhibition against topoisomerase II (Topo II) [8,9]. In continuation of searching for novel anticancer agents, we have synthesized a number of the ASP-A derivatives and evaluated for their anti-proliferation activity. Among them, AS1041 was cytotoxic to a panel of cancer cell lines with comparable potency with its parent compound ASP-A [7], and our screen results showed that AS1041 was more sensitive to K562 cells. Therefore, we want to investigate the detailed cytotoxicity and the related mechanisms of AS1041.

In this study, we reported the cytotoxicity of AS1041 and explored the related mechanisms. AS1041 inhibited the proliferation, arrested the cell cycle, and induced apoptosis in K562 cells. The molecular mechanic studies showed that AS1041 inactivated phospho- extracellular signal-regulated kinase (P-ERK) but activated the phosphatidylinositol 3 kinase/protein kinase B/mammalian target of rapamycin (PI3K/AKT/mTOR) pathway. Our results suggested that AS1041 was a promising anticancer lead compound and had potential in anticancer agent research and development.

## 2. Results and Discussion

### 2.1. Anticancer Spectrum of AS1041

To evaluate the cytotoxic effect of AS1041 on cancer cells, we first detected the proliferative inhibition rate of AS1041. As shown in Figure 1b, the half maximal inhibitory concentration (IC_50_) of AS1041 ranged from 1.56 to 10.30 μM, showing different cytotoxicity to various cancer cell lines, including K562, HeLa, HL-60, A549, CaSki, Jurkat, PC-3, Kasumi-1, MDA-MB-231, and BEL-7402 cell lines. However, AS1041 as high as 10 μM was not cytotoxic to other cells, including NCI-H1975, H22, Siha, and 4T1 (Figure 1c). Notably, compared with the other cancer cell lines, a marked anti-proliferative activity was observed in K562 cells, therefore, we selected the most sensitive K562 cells for the subsequent experiments.

### 2.2. AS1041 Inhibits the Proliferation of K562 Cells

**Figure 2:**
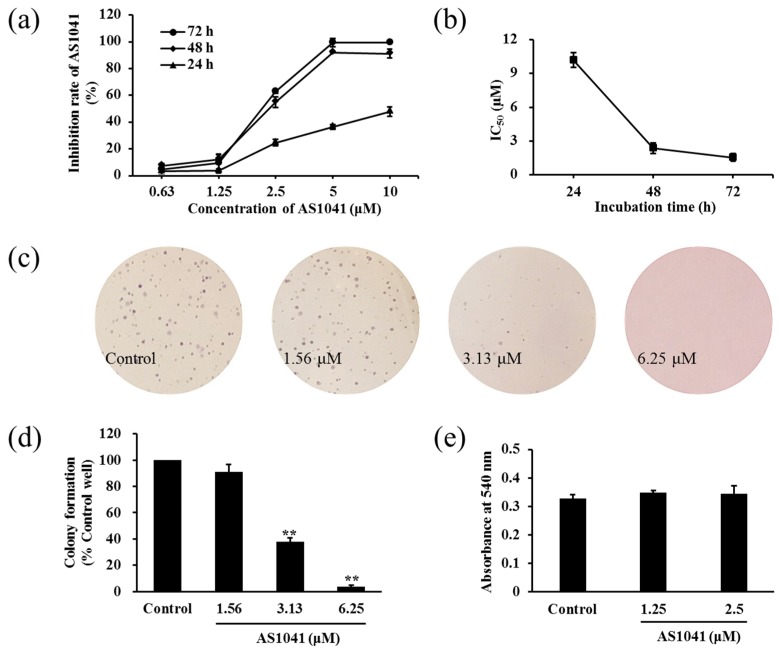
AS1041 inhibits K562 cells proliferation. (**a**) AS1041 inhibition rates (%) against K562 cells at different concentrations at 24, 48, and 72 h incubation. Cell viabilities were examined by the MTT assay. (**b**) IC_50_ values of AS1041 on K562 cells at 24, 48, and 72 h, respectively. (**c**) AS1041 inhibition on the formation of colonies in soft agarose. K562 cells were mixed in Dulbecco’s modified Eagle’s medium (DMEM) culture medium containing 0.35% agarose, in the absence or presence of different concentration of AS1041. Ten days later, the colonies were stained by crystal violet, then photos were taken, and numbered. (**d**) Quantification of the number of colonies in soft agarose. ** *p* < 0.01 vs. control. Data are presented as means ± SD from three independent experiments. (**e**) Effect of AS1041 on K562 cells differentiation. K562 cells were treated with indicated concentration of AS1041 for 72 h. Then, differentiation of K562 cells was measured by the nitroblue tetrazolium (NBT) reduction assay. Data are presented as mean ± SD for three independent experiments.

Since K562 cells were the most sensitive to AS1041, we evaluated the effect of AS1041 on K562 cells proliferation in detail. We found AS1041 inhibited the proliferation of K562 cells in a concentration- and time-dependent manner (Figure 2a). The IC_50_ values were 10.19, 2.37, and 1.56 μM at 24, 48, and 72 h, respectively (Figure 2b). The cellular proliferation inhibition was further confirmed by colony formation assay. As shown in Figure 2c, AS1041 significantly inhibited the formation and the diameter of the colonies, and the number of the colonies decreased in a concentration-dependent manner (Figure 2d), conforming the proliferation inhibition activities of AS1041 on K562 cells. Considering drug-induced malignant cell differentiation usually leads to the reduction in cell proliferation [10,11], and drug-induced cells differentiation is considered as a promising approach to treatment of leukemia [12], we then examined whether AS1041 inhibition on K562 cells proliferation had a relationship with differentiation, using nitroblue tetrazolium (NBT) reduction assay. The result showed that AS1041 did not affect the differentiation of K562 cells (*p* > 0.05, Figure 2e), indicating differentiation did not contribute to the proliferation inhibition in K562 cells. These results suggested that AS1041 inhibited K562 cells proliferation and was not via inducing cell differentiation.

### 2.3. AS1041 Induces Cell Cycle Arrest at G2/M Phase in K562 Cells

**Figure 3:**
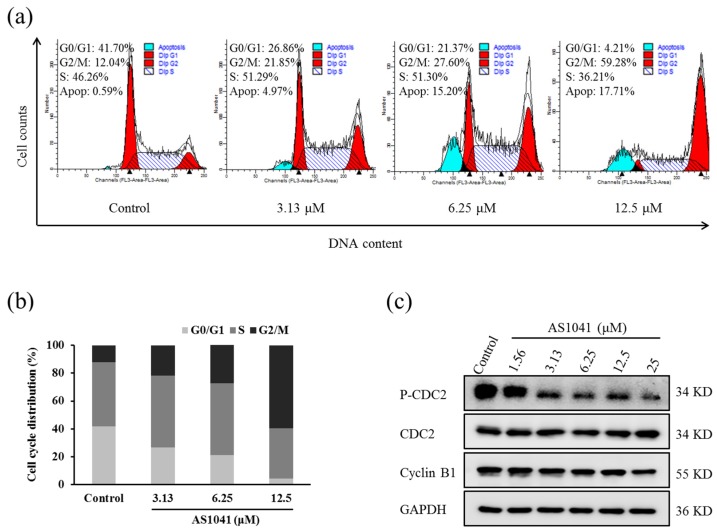
AS1041 arrests K562 cells in G2/M phase. (**a**) The cell cycle distribution of K562 cells exposed to AS1041 at different concentrations (0–12.5 μM) for 24 h. After treatment with AS1041, the cell were collected, washed, fixed, and stained by PI. Then, cells were detected by flow cytometry, and the data was analyzed with ModFit LT software. Different colors were used to distinguish the peaks corresponding to cells in apoptosis, G0/G1, S, and G2/M phase. (**b**) The bar graph depicts the percentage of each cell cycle phase of K562 cells in the absence or presence of AS1041. (**c**) Effects of AS1041 on the expression of G2/M phase related proteins. K562 cells were treated with indicated concentration of AS1041 for 24 h, respectively, and the related proteins were detected by Western blotting.

To explore the mechanisms underlying the cytotoxic activity of AS1041 in K562 cells, we detected the cell cycle distribution by flow cytometry, as in certain situations, induction of proliferation inhibition is a result of cycle arrest. As shown in Figure 3a, after AS1041 treatment, there was a decrease in the percentage of cells in G0/G1 phase and a significant increase in G2/M phase, in a concentration-dependent manner. The percentage of G2/M phase in the control group was 12.04%, and it increased to 21.85%, 27.60%, and 59.28%, when treated with 3.13, 6.25, and 12.5 μM AS1041, respectively (Figure 3b). Cyclin B1 and cell division cycle protein 2 (CDC2) are two key regulators of mitosis. Cyclin B1 associates with centrosomes and the mitotic spindle during mitosis, while CDC2 is a cell division cycle kinase and the activation of CyclinB1/CDC2 is generally considered to trigger the entry to mitosis [13,14]. Inhibiting CDC2 phosphorylation and downregulating Cyclin B1 usually lead to the cell cycle arrested at G2/M checkpoint [15,16]. After AS1041 treatment, the expression of Cyclin B1 decreased, and the activation of phospho-CDC2 (P-CDC2) decreased in a concentration-dependent manner (Figure 3c), confirming the G2/M phase arrest occurred after AS1041 treatment in K562 cells. These results indicated that AS1041 could arrest K562 cells at G2/M phase in a concentration-dependent manner. This was the same to other clinical chemotherapy agents, such as etoposide, which all inhibit the growth of cancer cells by inducing G2/M phase arrest [17].

Cell cycle regulation analysis has been vastly used as an important parameter to indicate apoptosis by detecting the appearance of cells in sub-G0/G1. When we detected the cell cycle distribution of K562 cells, we also found that the sub-G0/G1 phase cells appeared in a concentration-dependent manner after AS1041 treatment (Figure 3a). The percentage of sub-G0/G1 cells in the control group was 0.59%, and it increased to 4.97%, 15.20%, and 17.71% after treatment with AS1041 (3.13–12.5 μM), respectively, indicating AS1041 also possibly induced cell apoptosis.

### 2.4. AS1041 Induces Apoptosis in K562 Cells

**Figure 4:**
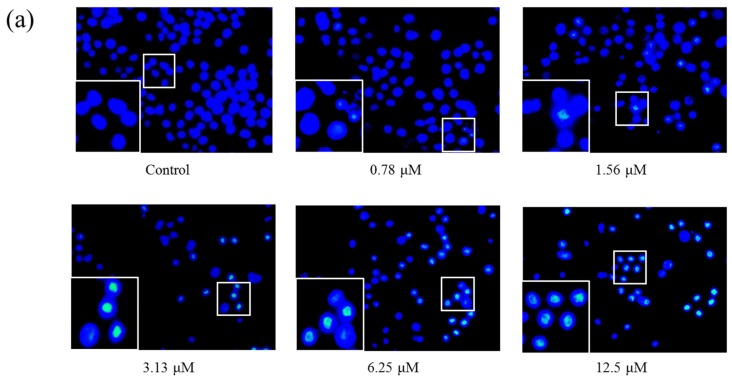

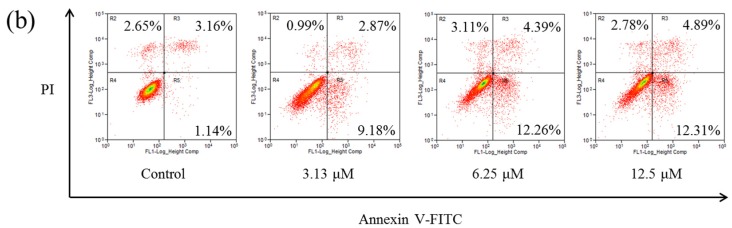
AS1041 induces apoptosis in K562 cells. (**a**) Changes in K562 cells nuclear morphology after AS1041 treatment. K562 cells were treated with AS1041 (0–12.5 μM) for 24 h, stained with Hoechst 33342, and visualized by fluorescence microscope. (**b**) Apoptosis in K562 cells induced by AS1041. K562 cells were treated with indicated concentration of AS1041 for 24 h, respectively, stained with Annexin V-fluorescein isothiocyanate (Annexin V-FITC)/PI, and determined by flow cytometry. R2 represents the necrosis cells, R3 represents the late apoptosis cells, R4 represents the normal cells, and R5 represents the early apoptosis cells.

In apoptosis, the nucleus undergo a series of changes including segregation of nucleoli and condensation of chromatin [18]. These events are considered to be the “gold standard” for detection of apoptosis [19]. To confirm AS1041 could induce apoptosis, we first performed Hoechst 33342 staining assay. As shown in Figure 4a, cell nucleus in the control group were round and stained weakly, while nucleus in cells treated with AS1041 became brighter, confirming chromatin condensation and apoptosis induction. Phosphatidylserine is locates on the inside of lipid bilayer, however, the phosphatidylserine exposes on the outside of the membrane when apoptosis occurred [20]. Annexin V shows high affinity to phosphatidylserine, and fluorochrome-conjugated annexin V and propidium iodide (PI) could distinguish and quantitatively analyze apoptotic cells [21]. Then, we detected phosphatidylserine outside of the membrane by Annexin V-fluorescein isothiocyanate (Annexin V-FITC)/PI double staining assay. As shown in Figure 4b, both the early (R5 region) and late (R3 region) stages of apoptosis cells were increased after AS1041 treatment. The total apoptotic cell percentage (the sum of R3 and R5 regions) of the control group was about 4.30%, and it increased to 12.05%, 16.65%, and 17.20% after treated with AS1041 (3.13–12.5 μM) for 24 h, respectively. These results revealed that AS1041 induced apoptosis in K562 cells. Thus, both cell cycle arrest and apoptosis induction were involved in inhibition of AS1041 against K562 proliferation, which was the same to most of the clinical anticancer chemotherapy agents. 

### 2.5. AS1041 Triggers Non-Caspase-Dependent Apoptosis in K562 Cells

**Figure 5:**
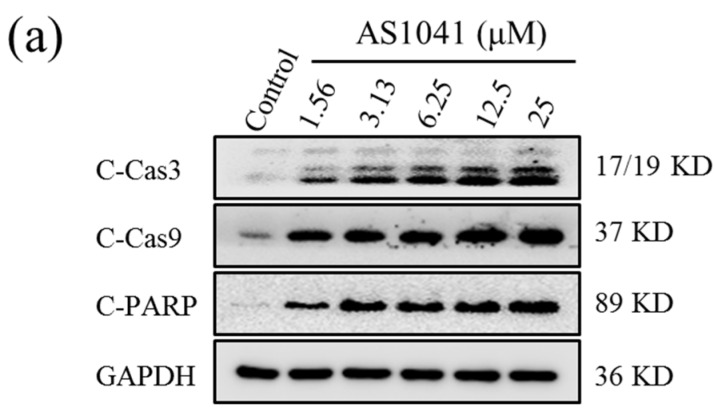

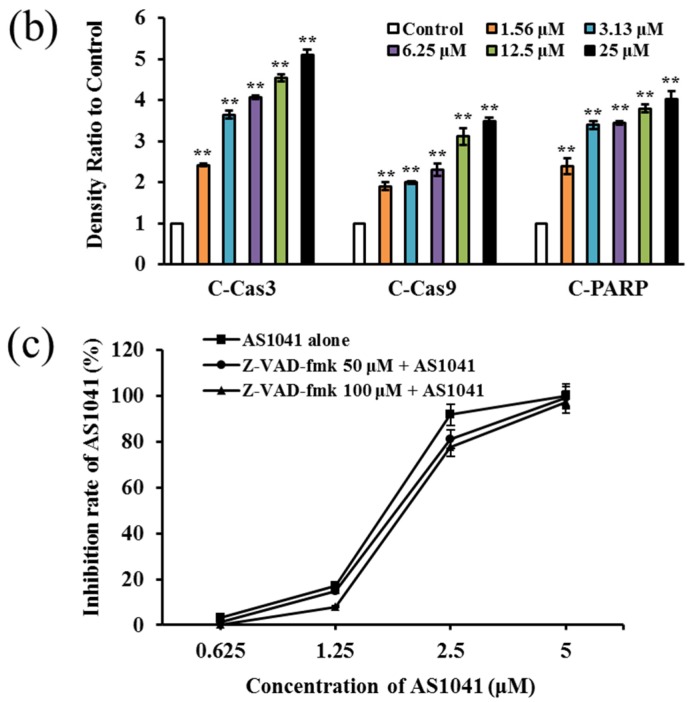
AS1041 triggers non-caspase-dependent apoptosis. (**a**) Effect of AS1041 on apoptosis-related proteins in K562 cells. K562 cells were treated with indicated concentration of AS1041 for 24 h, and then the cells were collected, washed, and lysed. Cleaved caspase-3 (C-Cas3), cleaved caspase-9 (C-Cas9), and cleaved Poly-adenosine diphosphate-ribose polymerase (C-PARP) were measured by Western blotting. (**b**) Histograms show the relative intensity of C-Cas3, C-Cas9, and C-PARP bands to control. Data are presented as mean ± SD for three independent experiments. ** *p* < 0.01 vs. control. (**c**) Pan-caspase inhibitor N-benzyloxycarbonyl-Val-Ala-Asp (OMe)-fluoromethylketone (Z-VAD-FMK) does not attenuate inhibition rate (%) of AS1041 on K562 cells. K562 cells were pre-treated with Z-VAD-FMK for 1 h, and then AS1041 for 72 h. K562 cells proliferation was measured by MTT assays. Data are presented as mean ± SD for three independent experiments.

Cysteinyl aspartate specific proteinase-9 (caspase-9) is the apical caspases in the intrinsic apoptosis pathways, and cysteinyl aspartate specific proteinase-3 (caspase-3) is considered to be the most important of the effector caspases. Cleaving and activation of the caspase-9/caspase-3 is a hallmark of the intrinsic apoptosis [18,22]. Poly-adenosine diphosphate-ribose polymerase (PARP) protein is a nuclear enzyme. Cleaved PARP (C-PARP) seems to be an early marker of apoptosis in cells, and increases in apoptosis cells [23]. We found that cleaved caspase-3 (C-Cas3), cleaved caspase-9 (C-Cas9), and C-PARP significantly increased after AS1041 treatment (Figure 5a), in a concentration-dependent manner (Figure 5b), confirming the apoptosis occurred after AS1041 treatment in K562 cells. However, pan-caspase inhibitor N-benzyloxycarbonyl-Val-Ala-Asp (OMe)-fluoromethylketone (Z-VAD-FMK) could not attenuate AS1041-induced cytotoxicity in K562 cells (Figure 5c), suggesting caspases were activated in AS1041-induced apoptosis but not dependent. Caspases have been recognized as important mediators of apoptosis, however, accumulating evidences indicating the existence of caspases-independent apoptosis [24]. Recently, a number of caspase-independent cell death factors and xenobiotics were reported to trigger caspase-independent apoptosis through different mechanisms and signaling pathways [25,26]. All these results strongly suggested that AS1041 could induce non-caspase-dependent apoptosis in K562 cells, and also indicated that other apoptosis pathways were also possibly involved in the apoptosis stimulation. 

### 2.6. AS1041 Localizes in the Cytoplasm and Does Not Inhibit the Topoisomerases

**Figure 6:**
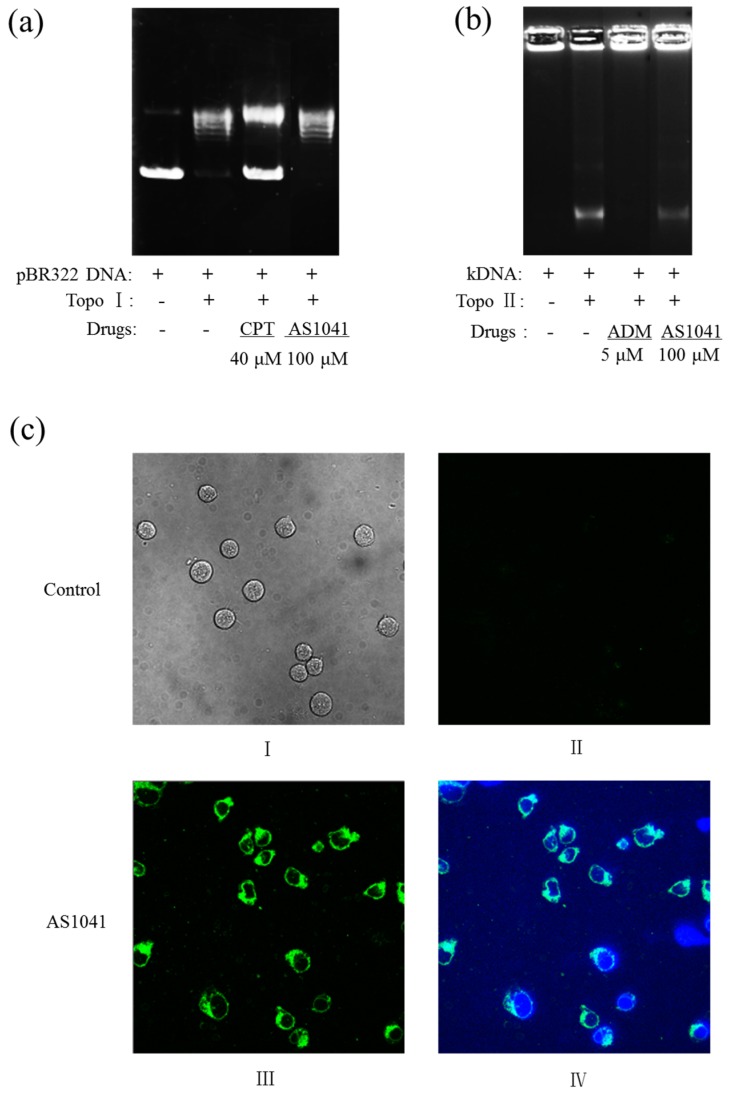
AS1041 localizes in the cytoplasm and does not inhibit the topoisomerases. (**a**) Inhibition effect of AS1041 on topoisomerase I (Topo I)-mediated relaxation of supercoiled plasmid pBR322 DNA. Plasmid pBR322 DNA was incubated at 37 °C for 30 min with or without Topo I in the presence of the indicated AS1041 (100 μM) and camptothecin (CPT, 40 μM) was used as a Topo I positive control inhibitor. (**b**) Inhibition effect of AS1041 on topoisomerase II (Topo II)-mediated kinetoplast DNA (kDNA) decatenation. kDNA was incubated at 37 °C for 30 min with or without Topo II in the presence of AS1041 (100 μM) and positive control adriamycin (ADM, 5 μM). (**c**) AS1041 localizes in the cytoplasm of K562 cells. Untreated K562 cells were taken photos using confocal microscopy (400×) in the bright field (I) and in 488 nm (AS1041 spontaneous fluorescence excitation wavelength) green fluorescence (II). AS1041-treated K562 cells were stained with DAPI, and were taken photos using a confocal microscopy in green fluorescence (488 nm) only (III), or in both green and blue fluorescence (IV).

Our previous work showed that ASP-A induced apoptosis via inhibition against Topo II [9], so we examined whether AS1041 also inhibited topoisomerases activity. We first detected the effects of AS1041 on topoisomerase I (Topo I) activity by Topo I-mediated relaxation of pBR322 DNA assay. As shown in Figure 6a, pBR322 DNA migrated slower when Topo I converted pBR322 to relaxed DNA, and supercoiled plasmid pBR322 DNA migrated faster when positive control camptothecin (CPT) inhibited the activity of Topo I. pBR322 DNA supercoil was untied in the presence of AS1041, showing that Topo I activity was not inhibited. We also examined the effects of AS1041 on Topo II activity by Topo II-mediated kinetoplast DNA (kDNA) decatenation assay. As shown in Figure 6b, the kDNA remained as very large reticulate DNA that could not migrate in agarose, and the kDNA became mini-circles which could migrate when Topo II added. kDNA remained as very large reticulate DNA and could not migrate when positive control adriamycin (ADM) inhibited the activity of Topo II. In the presence of AS1041, kDNA became mini-circles, indicating that Topo II activity was not inhibited. Although with no further experimental evidence, our preliminary molecule flexible docking studies showed that aromatic ring accumulation and hydrogen-bond interaction was the dominant force in the compounds (AS1041 and ASP-A) and Topo II interaction (data not shown). However, compared with ASP-A, which contains five phenolic hydroxyl groups, hydroxyl reduced derivatives, such as AS1041, showed lessened force binding to Topo II, though AS1041 showed intensive aromatic ring accumulation compared with ASP-A. We predicted that hydrogen-bond interaction between phenolic hydroxyl groups and Topo II was the dominant force among ASP-A, which was reduced in AS1041. We found AS1041 had a spontaneous fluorescence, which could be detected using excitation and emission wavelengths of 488 nm and 590 nm, and we further assayed the cellular localization of AS1041 in K562 cells. As shown in Figure 6c, in the absence of AS1041, K562 cells in the bright field (I) showed no fluorescence when excited at 488 nm (II), however, in the presence of AS1041, obvious green autofluorescence of AS1041 was observed (III), and this AS1041-originated fluorescence localized in the cytoplasm rather than the 4′,6-diamidino-2-phenylindole (DAPI)-stained nucleus (IV), indicated that AS1041 did not enter the nucleus, further confirming that AS1041-mediated apoptosis was not via topoisomerases inhibition, which was different from the parent compound ASP-A [9]. We speculated that other molecular mechanisms were involved in AS1041-induced apoptosis.

Derivatives of other kinds of compounds also have different action mechanisms to their parent compounds. For example, some genistein derivatives induce cell death and cell cycle arrest through different mechanisms to its parent compound genistein [27]. Cisplatin derivatives were also reported to exert biological activity via different mechanisms and, thus, show different cytotoxicity in most common cancer lines [28]. Structural modification of the parent compounds not only improves the bioactivity of the derivatives, but also results in producing different mechanisms of actions than the parent compound [29], which will be beneficial to the discovery of novel action mechanisms or targets of the new derivatives.

### 2.7. AS1041 Affects the Survial Signaling

**Figure 7:**
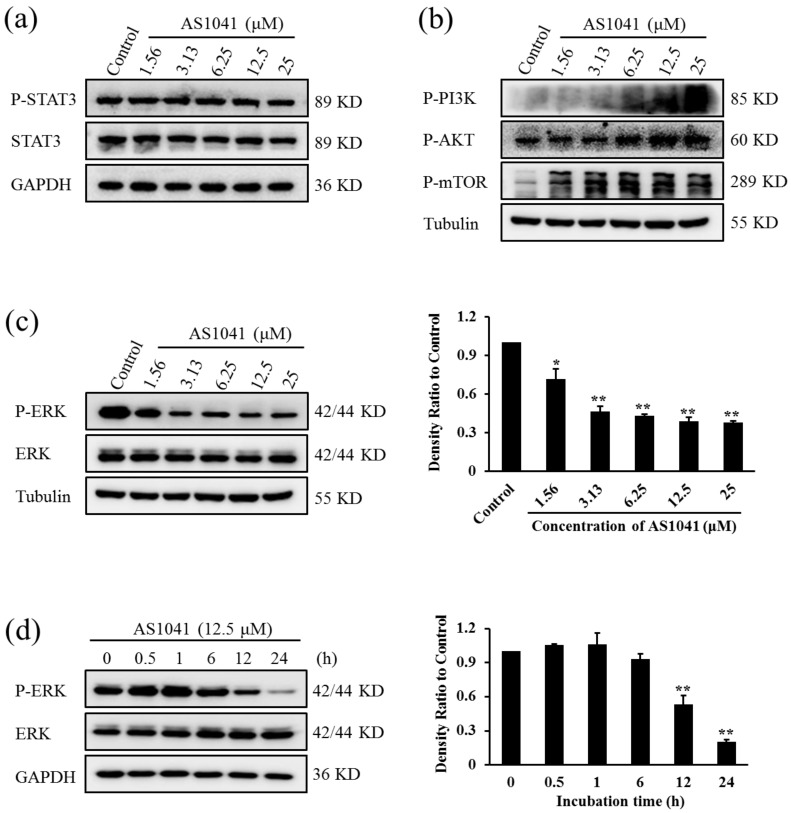
AS1041 inhibits the activation of ERK. (**a**) Effect of AS1041 on P-STAT3 pathways. K562 cells were treated with indicated concentration of AS1041 for 24 h. The protein levels of P-STAT3 and STAT3 were detected by Western blotting. (**b**) Activation of PI3K/AKT/mTOR pathway after treatment of AS1041. K562 cells were treated with indicated concentration of AS1041 for 24 h. The protein levels were detected by Western blotting. (**c**,**d**) P-ERK inactivation by AS1041 in K562 cells. K562 cells were treated with indicated concentration of AS1041 (**c**) or indicated time (**d**), protein levels were detected by Western blotting. Histograms show the relative intensity of P-ERK bands. Data are presented as mean ± SD for three independent experiments. * *p* < 0.05, ** *p* < 0.01 vs. control.

To illustrate the molecular mechanisms underlying AS1041 activity, we detected the changes on several key survival signal molecules in K562 cells. The signal transducer and activator of transcription 3 (STAT3) is one member of the signal transducers, and is activated by cytokines and growth factors [30]. Activation of STAT3 has been reported to promote the proliferation and survival in various cells, while inhibition of STAT3 could induce apoptosis in cancer cells [31,32]. We then examined whether AS1041 inhibited STAT3 phosphorylation. As shown in Figure 7a, AS1041 did not inhibit the activation of STAT3, suggesting STAT3-mediated signaling pathways were not affected. Inhibition on PI3K/AKT/mTOR pathway is a vital way to inhibit the proliferation of leukemia and many anticancer agents induce apoptosis via inhibiting this signal pathway [33,34]. However, in the case of AS1041, PI3K/AKT/mTOR signaling pathway was unexpectedly activated (Figure 7b), indicating AS1041-induced apoptosis was not via inhibition on PI3K/AKT/mTOR pathway. Activating extracellular signal-regulated kinase (ERK) pathway usually promotes proliferation of human cancer cells and inhibition activation of ERK will triggers apoptosis [35,36]. In our present work, we found that activation of P-ERK was significantly inhibited after AS1041 treatment, without any effect on the total protein of ERK, and the inhibition was both in a concentration- and time-dependent manner (Figure 7c,d). In the treatment of leukemia using imatinib, higher activation of P-ERK is usually observed and this activation of ERK usually led to drug resistance [37,38]. AS1041 could decrease the level of P-ERK, suggesting that AS1041 may potentiate the anticancer effect of imatinib in K562 cells. As shown in Table 1, when combined with imatinib and AS1041, all the CI values were smaller than 1, indicating the potent synergism between AS1041 and imatinib on the inhibition of K562 cells proliferation and the chemosensitizing effect of AS1041. Moreover, this result further confirmed AS1041 inactivated ERK in K562 cells. All these results indicated that AS1041 induced K562 cells apoptosis by inhibition of P-ERK activation. This was similar with other compounds, such as nitidine chloride, which also inhibited the activation of ERK and induced apoptosis [39]. This inhibition on ERK and induction of apoptosis may explain why AS1041-induced apoptosis is not caspase dependent. 

A number of anticancer agents inactivate the PI3K/AKT/mTOR signaling pathway and subsequently inhibit cell proliferation and induce apoptosis [40]. However, as a response and feedback to inhibition on other signaling molecules and the apoptosis, AKT sometimes also activates to enhance the cell proliferation and survival [41]. This kind of AKT activation was considered to contribute to drug resistance and reduce response to chemotherapeutic agents [42]. This feedback activation of PI3K/AKT/mTOR is often observed in mitogen-activated protein kinase (MAPK)/ERK kinase (MEK) inhibitor-induced ERK inhibition [43,44]. Feedback activation of PI3K/AKT/mTOR pathway decreases the susceptibility of cancer cells to MEK/ERK inhibition [45,46,47]. We hypothesized that AS1041-induced activation of PI3K/AKT/mTOR pathway possibly was also a feedback for the cytotoxicity of AS1041 and inhibition of ERK, and played a protective role against AS1041 damage. Our present work supported the conception that interplays between EKR/AKT oncogenic pathways potentially impact the sensitivity of cancers to their inhibitors [43]. However, our hypothesis needs furthermore experiments to be proved, for example, combination use of PI3K/AKT/mTOR inhibitors with AS1041 to evaluate whether these inhibitors could increase the efficiency of AS1041. To the best of our knowledge, this is the first report that revealing ASP-A derivative could simultaneously inactivate ERK and activate PI3K/AKT/mTOR. 

## 3. Conclusions

In conclusion, our present study provided solid evidence that AS1041 could inhibit the proliferation of K562 cells, which resulted from both cell cycle arrest and apoptosis. Unlike its parent compound ASP-A, AS1041 did not inhibit the activity of topoisomerases. In addition, for the first time, we revealed that AS1041 could activate PI3K/AKT/mTOR pathway and inactivate ERK. The inhibition against ERK contributed to AS1041 induced apoptosis and sensitized imatinib. Our present study suggests that AS1041 is a promising lead compound for the chemotherapeutic or chemosensitizing agent development.

## 4. Materials and Methods

### 4.1. Reagents and Drug

Compound AS1041 were provided by Key laboratory of Marine Drugs, the Ministry of Education of China, School of Medicine and Pharmacy, Ocean University of China. The chemical structure of AS1041 was identified by ^1^H nuclear magnetic resonance (NMR), ^13^C NMR, and high resolution mass spectrum (HRMS) [7]. Antibodies against C-Cas3, C-Cas9, C-PARP, P-CDC2, CDC2, Cyclin B1, P-ERK, ERK, P-STAT3, and STAT3 were purchased from Cell Signaling Technology (Boston, MA, USA). Annexin V-FITC apoptosis detection kit was purchased from KeyGEN BioTECH. Co., Ltd, Nanjing, China. The kinetoplast DNA (kDNA) and Topoisomerase II α human (Topo II) were from TopoGEN (Port Orange, FL, USA) and Sigma-Aldrich (St. Louis, MO, USA). Plasmid pBR322 DNA and DNA topoisomerase I (Topo I) was purchased from TaKaRa (Dalian, China). GelRedTM Nucleic Acid Gel Stain, 10,000× in water was from MDBio Inc. Other reagents and kits were from Beyotime Biotechnology, China.

### 4.2. Cell Lines and Cell Culture

Human chronic myelogenous leukemia K562 cells, human promyelocytic leukemia HL-60 cells, human acute T lymphocytic leukemia Jurkat cells, human acute myeloid leukemia cell line Kasumi-1, human cervical carcinoma HeLa cells, human cervical squamous carcinoma SiHa cells, human cervical cancer cell lines CaSki, human hepatocellular carcinoma BEL-7402, human lung adenocarcinoma cell line A549, human breast cancer MDA-MB-231, human prostate cancer PC-3, mouse hepatoma cell line H22, and murine breast carcinoma cell line 4T1 were obtained from Shanghai Cell Bank, Chinese Academy of Science. The human non-small cell lung cancer cell line NCI-H1975 was provided by the American Type Culture Collection. A549 cell line was cultured in Ham’s F12K medium (F12K) with 10% fetal bovine serum and 1% penicillin/streptomycin. The K562 cell line was cultured in Iscove’s Modified Dulbecco’s medium (IMDM) with 10% fetal bovine serum and 1% penicillin/streptomycin. HeLa cell line was cultured in modified Eagle’s medium (MEM) with 10% fetal bovine serum and 1% penicillin/streptomycin, and others were cultured in Roswell Park Memorial Institute (RPMI)-1640 medium with 10% fetal bovine serum and 1% penicillin/streptomycin. All the cells were cultured at 37 °C with 5% CO_2_ in a humidified incubator.

### 4.3. Cell Viability Assessment

Cell viability was assayed by 3-(4,5-dimethylthiazol-2-yl)-2,5-diphenyltetrazolium bromide (MTT) method or sulforhodamine B (SRB) test. For MTT assay, cells were seeded into 96-well plates and treated with gradient concentrations of AS1041 for 72 h. MTT (5 mg/mL, 20 μL) was added to each well and incubated for another 4 h, then the formazan product was dissolved with 150 μL dimethyl sulfoxide (DMSO) and detected at a wavelength of 570 nm using a microplate reader (BioTek, Winooski, VT, USA). For SRB assay, after adherent cells were treated, the medium was removed and the cells were fixed with 10% trichloroacetic acid at 4 °C for 1 h. After washing with water, SRB (100 μL) was added with 1% glacial acetic acid and incubated at room temperature for 15 min. The bound stain was solubilized with Tris buffer (150 μL) at 37 °C for 10 min, and optical density was measured at 515 nm using a microplate reader.

### 4.4. Cell Cycle Analysis

K562 cells were seeded in six-well plates (5 × 10^5^ cells each well), and treated with different concentrations of AS1041 for 24 h. The control group was treated with vehicle DMSO (also for other assays). Cells were collected, washed, and fixed in 70% cold ethanol overnight at −20 °C. Cells were collected and treated with ribonuclease (RNase) (25 μg/mL) for 1 h at 37 °C. Then cells were collected, washed, and stained with PI (100 μg/mL) for 30 min in the dark. The cell cycle distribution was detected with flow cytometry system (Beckman Coulter MoFlo XDP, Fullerton, CA, USA), and the data were analyzed with ModFit LT software (Verity Software House. Inc, Topsham, ME, USA).

### 4.5. Annexin V-FITC/PI Analysis Assay

K562 cells were seeded in six-well plates (5 × 10^5^ cells each well), and treated with different concentrations of AS1041 for 24 h. After AS1041 treatment, apoptosis was detected using an Annexin V-FITC apoptosis detection kit, according to the manufactures’ protocol. Briefly, cells were treated with different concentrations of AS1041, harvested and washed ice-cold phosphate buffered saline (PBS), and then resuspended in 100 μL binding buffer containing 5 μL Annexin V-FITC and 5 μL PI, which were all contained in the detection kit. After incubation for 10–15 min in the dark at room temperature, 400 μL binding buffer was added to the cell suspension. Cells were analyzed on an Aria FACS flow cytometry system (Beckman Coulter MoFlo XDP, Fullerton, CA, USA).

### 4.6. Hoechst 33342 Staining Assay

K562 cells were seeded in six-well plates (5 × 10^5^ cells each well), and cultured with different concentrations of AS1041 for 24 h. Hoechst 33342 was added to a final concentration of 5 μg/mL for 30 min. Cells were collected, washed, and resuspended in PBS. Nucleus morphology was examined by fluorescence microscope (OLYMPUS, Tokyo, Japan).

### 4.7. Western Blotting Assay

K562 cells were seeded in six-well plates (5 × 10^5^ cells each well), and treated with different concentrations of AS1041 or treated with different time. Cells were collected, washed, and lysed with proteins loading buffer (0.125 M Tris-HCl, 5% 2-Mercaptoethanol, 30 mg/mL sodium dodecyl sulfate (SDS), 10% glycerol, 0.5 mg/mL bromophenol blue) for 45 min on ice. Protein samples were separated on 7–15% SDS-polyacrylamide gels, and transferred to nitrocellulose membranes. The membrane was blocked and incubated with primary antibodies overnight at 4 °C, and then incubated with HRP-coupled secondary antibody at room temperature. Glyceraldehyde-3-phosphate dehydrogenase (GDPDH) or tubulin was used as the internal loading control. Specific proteins were detected with enhanced chemiluminescense by FluorChem E (Protein Simple, San Jose, CA, USA).

### 4.8. Soft Agar Colony Formation Assay

K562 cells colony-forming activity was assayed using low gelling temperature agarose. Prewarmed 2× Dulbecco’s modified Eagle’s medium (DMEM, 1 mL, containing 20% fetal bovine serum and 2% penicillin/streptomycin) and 1.2% bottom agarose solution (1 mL) were mixed and transferred into six-well plates. After the hardening of the bottom layer, K562 cells (3000 cells each well) were mixed with 2 mL culture medium containing 0.35% agarose and different concentration of AS1041, and then added to the plates as the upper layer. Ten days later, the colonies were stained with crystal violet, taken photos, and analyzed.

### 4.9. NBT Reduction Assay

K562 cells were seeded in six-well plates (5 × 10^5^ cells each well), and treated with or without different concentrations of AS1041 for 72 h. One million cells in each sample were collected, and washed. Then, cells were incubated with NBT (1 mg/mL) dissolved in PBS, containing 500 ng/mL PMA (phorbol 12-myristate 13-acetate), at 37 °C for 1 h in darkness. After washing with PBS, 1 mL 10% SDS was added with 0.04 mM HCl and incubated overnight at 37 °C. The above liquid was taken from 200 μL in each group and transferred to a 96-well plate to measure the absorbance at 540 nm using a microplate reader.

### 4.10. Topo I-Mediated Relaxation of pBR322 DNA Assay

A total of 20 μL reaction containing pBR322 DNA 0.5 μg, 2 μL 10 × DNA Topo I Buffer, 1 U Topo I, 2 μL 0.1% BSA, was incubated with or without the AS1041 at 37 °C for 30 min. CPT was used as a positive control. The reaction was terminated by adding 2 μL of DNA 10× loading buffer and subjected to electrophoresis in 1% agarose containing 1/10,000 GelRedTM Nucleic Acid Gel Stain. Gels were visualized by FluorChem E (Protein Simple, San Jose, CA, USA).

### 4.11. Topo II-Mediated kDNA Decatenation Assay

A total of 20 μL reaction containing 50 mM Tris, 120 mM KCl, 10 mM MgCl_2_, 0.5 mM ATP, 0.5 mM DTT, 30 μg/mL BSA, 200 ng kDNA, and 1 U Topo II was incubated with or without AS1041 at 37 °C for 30 min. ADM was used as a positive control. The reaction was terminated by adding 2 μL of DNA 10× loading buffer and subjected to electrophoresis in 1% agarose containing 1/10,000 GelRedTM Nucleic Acid Gel Stain. Gels were visualized by FluorChem E (Protein Simple, San Jose, CA, USA).

### 4.12. Intracellular Localization of AS1041 in K562 Cells

K562 cells were seeded in six-well plates (5 × 10^5^ cells each well), and treated with AS1041 (25 μM) for 2 h. Then, the cells were harvested and suspended in DAPI (10 μg/mL) at 37 °C for 10 min in the dark. AS1041 has a spontaneous fluorescence, which can be detected using 488 nm and 590 nm as excitation and emission wavelengths. K562 cells were observed by a confocal microscopy (ZEISS, Jena, Germany).

### 4.13. Statistical Analysis

The data shown in this study represented the mean values ± SD. Differences between the groups were assessed by Student’s *t*-test using Microsoft Office Excel (Microsoft, Redmond, WA, USA), and statistical significance was defined as *p* < 0.05.

## Figures and Tables

**Table 1:** AS1041 shows synergistic effect with imatinib. AS1041 shows synergistic effect with imatinib. K562 cells were treated with imatinib (0.06–1 μM) in the presence of AS1041 (1.56–6.25 μM) for 72 h. The combination index (CI) was calculated by Calcusyn (Biosoft, Cambridge, UK). The CI values of >1, 1, and <1 indicate antagonistic, additive, and synergistic effects, respectively.

AS1041 (μM)	Imatinib (μM)	CI
6.25	1.00	0.59
6.25	0.50	0.32
6.25	0.25	0.18
6.25	0.13	0.12
6.25	0.06	0.08
3.13	1.00	0.57
3.13	0.50	0.30
3.13	0.25	0.16
3.13	0.13	0.09
3.13	0.06	0.06
1.56	1.00	0.56
1.56	0.50	0.28
1.56	0.25	0.15
1.56	0.13	0.59
1.56	0.06	0.72

## Abbreviations

ASP-A, aspergiolide A; MTT, 3-(4,5-dimethylthiazol-2-yl)-2,5-diphenyltetrazolium bromide; SRB, sulforhodamine B; NBT, nitroblue tetrazolium; CDC2, cell division cycle protein 2; P-CDC2, phospho-CDC2; PI, propidium iodide; Annexin V-FITC, Annexin V-fluorescein isothiocyanate; PARP, Poly-adenosine diphosphate-ribose polymerase; C-PARP, cleaved PARP; caspase-3, cysteinyl aspartate specific proteinase-3; C-Cas3, cleaved caspase-3; caspase-9, cysteinyl aspartate specific proteinase-9; C-Cas9, cleaved caspase-9; Z-VAD-FMK, N-benzyloxycarbonyl-Val-Ala-Asp (OMe)-fluoromethylketone; Topo II, topoisomerase II; Topo I, topoisomerase I; CPT, camptothecin; kDNA, kinetoplast DNA; ADM, adriamycin; DAPI, 4′,6-diamidino-2-phenylindole; STAT3, signal transducer and activator of transcription 3; PI3K/AKT/mTOR pathway, phosphatidylinositol 3 kinase/protein kinase B/mammalian target of rapamycin pathway; ERK, extracellular signal-regulated kinase; P-ERK, phospho-ERK; MEK, mitogen-activated protein kinase (MAPK)/ERK kinase; CI, combination index; NMR, nuclear magnetic resonance; HRMS, high resolution mass spectrum; F12K, Ham’s F12K medium; DMEM, Dulbecco’s modified Eagle’s medium; IMDM, Iscove’s modified Dulbecco’s medium; MEM, modified Eagle’s medium; RPMI-1640 medium, Roswell Park Memorial Institute-1640 medium; DMSO, dimethyl sulfoxide; RNase, ribonuclease; PMA, phorbol 12-myristate 13-acetate; GAPDH, glyceraldehyde-3-phosphate dehydrogenase.
